# Influence of C-terminal truncation of murine Serum amyloid A on fibril structure

**DOI:** 10.1038/s41598-017-06419-1

**Published:** 2017-07-21

**Authors:** Matthies Rennegarbe, Inga Lenter, Angelika Schierhorn, Romy Sawilla, Christian Haupt

**Affiliations:** 1 Institute of Protein Biochemistry, Ulm University, Helmholtzstraße 8/1, 89081 Ulm, Germany; 2 Max Planck Research Unit for Enzymology of Protein Folding, Weinbergweg 22, 06120 Halle (Saale), Germany; 3 Institute of Biochemistry and Biotechnology, Martin-Luther-University, Kurt-Mothes-Straße 3, 06120 Halle (Saale), Germany

## Abstract

Amyloid A (AA) amyloidosis is a systemic protein misfolding disease affecting humans and other vertebrates. While the protein precursor in humans and mice is the acute-phase reactant serum amyloid A (SAA) 1.1, the deposited fibrils consist mainly of C-terminally truncated SAA fragments, termed AA proteins. For yet unknown reasons, phenotypic variations in the AA amyloid distribution pattern are clearly associated with specific AA proteins. Here we describe a bacterial expression system and chromatographic strategies to obtain significant amounts of C-terminally truncated fragments of murine SAA1.1 that correspond in truncation position to relevant pathological AA proteins found in humans. This enables us to investigate systematically structural features of derived fibrils. All fragments form fibrils under nearly physiological conditions that show similar morphological appearance and amyloid-like properties as evident from amyloid-specific dye binding, transmission electron microscopy and infrared spectroscopy. However, infrared spectroscopy suggests variations in the structural organization of the amyloid fibrils that might be derived from a modulating role of the C-terminus for the fibril structure. These results provide insights, which can help to get a better understanding of the molecular mechanisms underlying the different clinical phenotypes of AA amyloidosis.

## Introduction

Amyloidosis is a group of diseases associated with the pathological deposition of amyloid fibrils in the body, including Alzheimer’s and prion diseases, type II diabetes or different systemic amyloidosis^1^. Systemic AA amyloidosis occurs in humans, other mammals and birds and is defined by the deposition of amyloid fibrils derived from serum amyloid A (SAA) 1.1 protein^2^. Deposits can be found in different organs in particular kidney, spleen, liver and gastrointestinal tract. Kidney amyloid severely damages the native organ functions and causes proteinuria that is one of the first clinical symptoms of the disease^3^.

SAA1.1 protein has a length of 103 amino acids in the mouse and of 104 amino acids in humans^1^. A great similarity exists between human and murine genes including sequence homology, the boundary position of exons and introns and the presence of several SAA polymorphisms^4, 5^. These polymorphisms result only in small differences in amino acid sequence and have been described and summarized in detail elsewhere^1, 6^. Within a larger protein family, only SAA1.1 contributes to amyloidosis in humans and mice^1^. It is a blood-borne acute phase apolipoprotein that normally occurs at concentrations of 1–3 μg/ml. Strong inflammatory stimuli lead to a dramatic upregulation of its hepatic production, and protein concentrations eventually reach levels of >1 mg/ml in the blood. SAA1.1 occurs within the high-density lipoprotein (HDL) 3 fraction^7^. The atomic structures of human SAA1.1 and murine SAA3 are known and show striking similarities with pronounced α-helical secondary structure^8, 9^. The murine SAA1.1 structure has not been solved yet, however it also possesses high α-helical content, indicating similar underlying structure^10^.

The major protein species found in AA amyloid is practically never full-length SAA but instead C-terminally truncated fragments, termed AA proteins^11, 12^. Interestingly, different AA proteins are clearly associated with phenotypic variations in the AA amyloid distribution pattern. The most commonly reported AA protein is SAA(1–76), which frequently deposits in the renal glomeruli^13^. A rarer variant is associated with SAA fragments terminating at residues 45 and 85 that tend to accumulate in the renal medulla and blood vessel walls instead of the glomeruli^13^. In addition, a spectrum of other SAA fragments has been shown to occur in diseased tissue, for example 82 and 94 amino acid residues which were found in patients with heavy vascular amyloid infiltration^14^. Recently a central role of matrix metalloproteinases (MMP) has been proposed in pathogenesis of AA amyloidosis. Cleavage of SAA with MMP’s results in N-terminal fragments of mainly SAA(1–57)^15, 16^. The reasons for the different distribution patterns of AA proteins and the existence of clinical phenotypes are completely unknown. However, the properties of the AA proteins have never been systematically investigated and there has been long-standing debate as to whether C-terminal cleavage occurs before or after amyloid formation.

Here, we describe a recombinant expression system that enables us to obtain large quantities of C-terminally truncated fragments of full-length murine SAA1.1. This allows us to systematically investigate for the first time the influence of C-terminal truncation of murine serum amyloid A on the structural features of the derived fibrils to get insights into the mechanism of pathology of AA amyloidosis.

## Results

**Figure 1:**
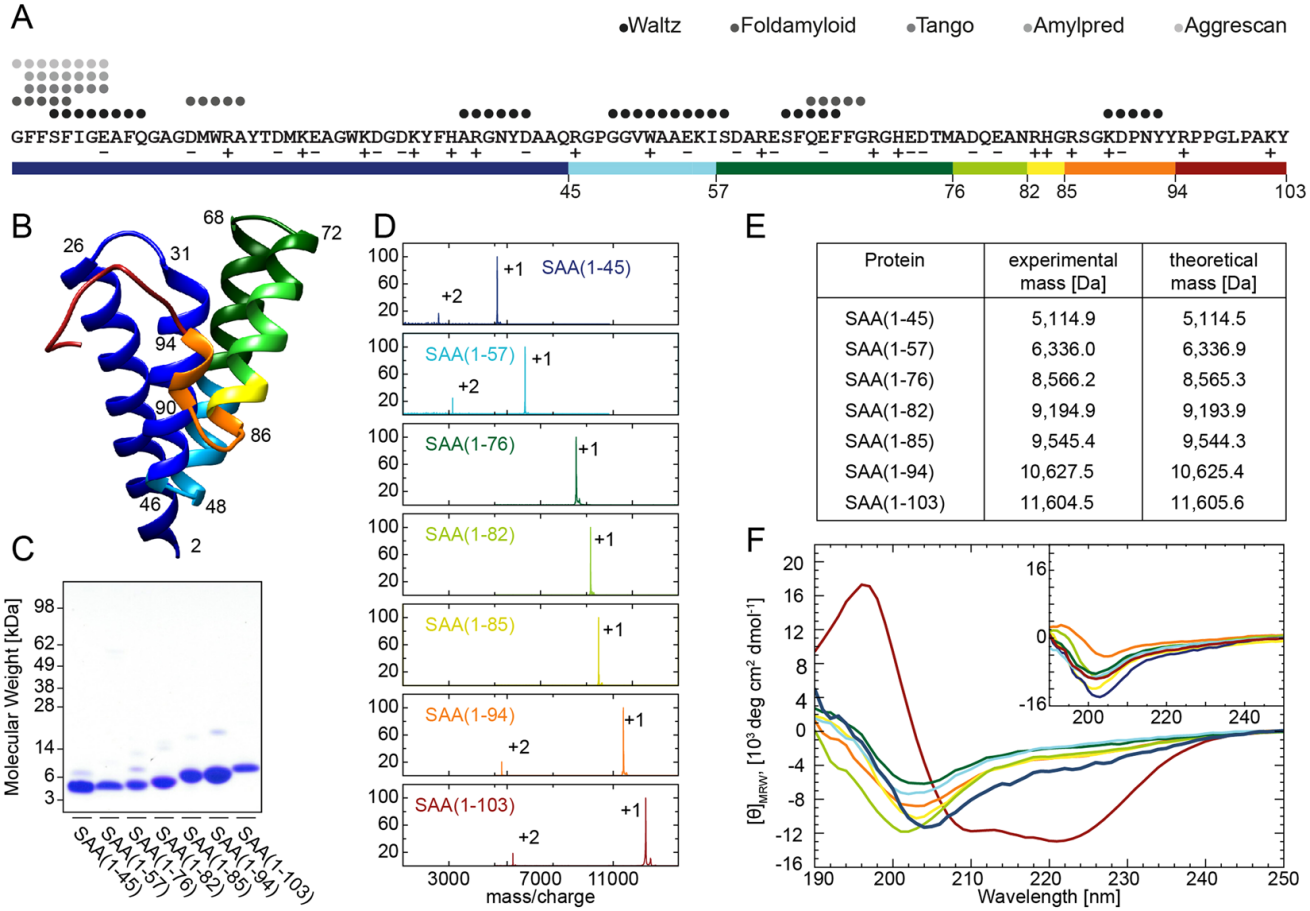
Properties of SAA(1–103) and its AA proteins. (**A**) Residue-specific prediction of amyloid prone regions within murine SAA1.1 protein using five different algorithms as indicated in the panel. Investigated fragments are marked in colors. Red, SAA(1–103); orange, SAA(1–94); yellow, SAA(1–85); light green, SAA(1–82); dark green, SAA(1–76); light blue, SAA(1–57); dark blue, SAA(1–45). Acidic amino acid residues are labeled with plus and basic residues with minus. (**B**) Ribbon representation of a homology model of murine SAA1.1 based on the crystal structure of human SAA1.1^9^ (PDB:4IP9). First and last residues of helical secondary structures are labeled in the panel (**C**) Coomassie stained SDS-PAGE of recombinantly produced SAA(1–103) and AA proteins. (**D**) MALDI mass spectra of SAA(1–103) and SAA fragments. Peaks labeled with +1 represent the [M+H]^+^ ions while peaks labeled with +2 represent the [M+2H]^2+^ ions. (**E**) Masses of SAA(1–103) and SAA fragments determined by mass spectrometry in comparison to theoretical masses. (**F**) Far-UV CD spectra of SAA(1–103) and investigated fragments at 4 °C and 37 °C (insert). (**A**,**B**,**D** and **F**) use the same color coding.

We started with a theoretical analysis of intrinsic properties of full-length murine SAA, termed here SAA(1–103) and its C-terminally truncated fragments SAA(1–45), SAA(1–57), SAA(1–76), SAA(1–82), SAA(1–85) and SAA(1–94) (Fig. 1A). These fragments were chosen because of their high similarity to fragments found in diseased human tissues. First, we analyzed the charge distribution of the amino acid sequence of SAA(1–103). It has a comparable number of acidic and basic amino acid residues resulting in an overall net charge of −1.9 under physiological conditions. The isoelectric point (pI) of the full-length protein is 5.89 (Table 1). Even though the charged amino acids are evenly distributed throughout the sequence, there is a notable accumulation of positively charged residues within the last 21 amino acids of SAA(1–103). Removal of the C-terminus results in a decrease of the pIs up to SAA(1–82), which bears the most negative net charge (−6) and consequently has the lowest pI of 4.69. SAA(1–57) stands out with the smallest net charge of −1.1 and a pI of 5.63, resembling the characteristics of SAA(1–103). This may indicate that SAA(1–103) and SAA(1–57) possess the lowest solubility at near physiological pH of all proteins examined. We further analyzed the amino acid sequence of SAA(1–103) with five different algorithms to predict aggregation prone regions (Fig. 1A). Strikingly, all algorithms detect an amyloidogenic hotspot within the first 11 amino acids. This N-terminal sequence has been shown to be highly important for the fibrillation of SAA^17, 18^. In addition, two algorithms also found amyloid prone regions between amino acids 15–19, 37–42, 49–58, 63–69 and 89–93. Interestingly, many of these regions are located next to the C-terminus of the fragments chosen in this study (Fig. 1A). This suggests that not only the N-terminal region but also the amino acid sequence forming the C-terminus of the truncated peptides may have an influence on the fibril structure. All predicted regions are located in the helices of the globular SAA structure as evident from a homology model based on the crystal structure of human SAA1.1^9^ and the amino acid sequence of SAA(1–103) (Fig. 1B). This suggests that these regions are protected against aggregation, as long as the helix-bundle fold is maintained, which was proposed to be highly dependent on the presence of the C-terminal tail of SAA^9^.

**Table 1:** Theoretical analysis of the amino acid sequences of SAA(1–103) and AA proteins.

Polypeptide	Number of basic amino acid residues	Number of acidic amino acid residues	Net charge at pH 7.4	Theoretical pI
SAA(1–103)	17	16	−1.9	5.89
SAA(1–94)	15	16	−3.9	5.26
SAA(1–85)	13	15	−4.9	5.02
SAA(1–82)	11	15	−6.0	4.69
SAA(1–76)	11	13	−4.0	4.97
SAA(1–57)	8	8	−1.1	5.63
SAA(1–45)	6	7	−2.1	4.95

Next, we have established bacterial expression systems and chromatographic strategies to generate and purify chosen AA proteins. Based on an already available expression system for the soluble expression of SAA(1–103)^19^ we generated new expression vectors and established cultivation conditions for the production of the relevant AA proteins. Although the bacterial expression of certain SAA isoforms and fragments has been shown from different laboratories^9, 19–21^, to our knowledge this is one of the first reports on the soluble expression of murine SAA1.1 fragments that correspond to AA proteins found in diseased tissue. The established expression systems enable us to obtain between 5 mg (SAA(1–45)) and 47 mg (SAA(1–94)) of purified peptide from 50 g wet cell pellet. Denaturing gel electrophoresis of the purified peptides shows the presence of a major protein species between roughly 11 kDa and 4 kDa and only few impurities (Fig. 1C). To verify the chemical identity of the peptides we used mass spectrometry. All detected peaks represent either the single or double charged ions of the respective peptide, supporting the conclusion that the samples are highly pure (Fig. 1D). The determined masses correspond well to the theoretical masses expected from the amino acid sequence (Fig. 1E).

The absence of the C-terminal tail of SAA(1–103) in the truncated proteins suggests that the structural integrity of the 4-helix bundle might be disturbed. To analyze the secondary structure of the AA proteins in solution, we freshly dissolved the peptides in buffer and carried out far-UV circular dichroism (CD) spectroscopy at 4 °C and 37 °C. As reference we first analyzed the secondary structure of SAA(1–103) at 4 °C which has already been shown to be α-helical^10, 21^. The CD spectrum of SAA(1–103) at 4 °C showed one single maximum around 197 nm and two minima centered around 208 and 222 nm, which indicates the presence of a largely α-helical secondary structure^22^ (Fig. 1F). In contrast, at 37 °C SAA(1–103) shows only a single minimum centered around 202 nm, which suggests a significant loss in α-helical secondary structure. Analysis of the freshly dissolved AA proteins revealed that none of them possesses a pronounced α-helical secondary structure, neither at 4 °C nor at 37 °C. Instead, all spectra showed a single minimum at around 200–204 nm, closely resembling the CD spectrum of SAA(1–103) at 37 °C. This indicates that the fragments possess a significantly less amount of well-ordered secondary structure compared to the full-length protein. These data show that the absence of the C-terminal tail of SAA(1–103) (residues 95–103) is sufficient to cause a significant loss in α-helical secondary structure of the otherwise stable globular fold at 4 °C. Based on this result we conclude that the amyloidogenic regions of the investigated AA proteins are highly exposed at physiological temperature, which let us assume that all of the C-terminally truncated fragments of SAA(1–103) are able to form amyloid fibrils.

**Figure 2:**
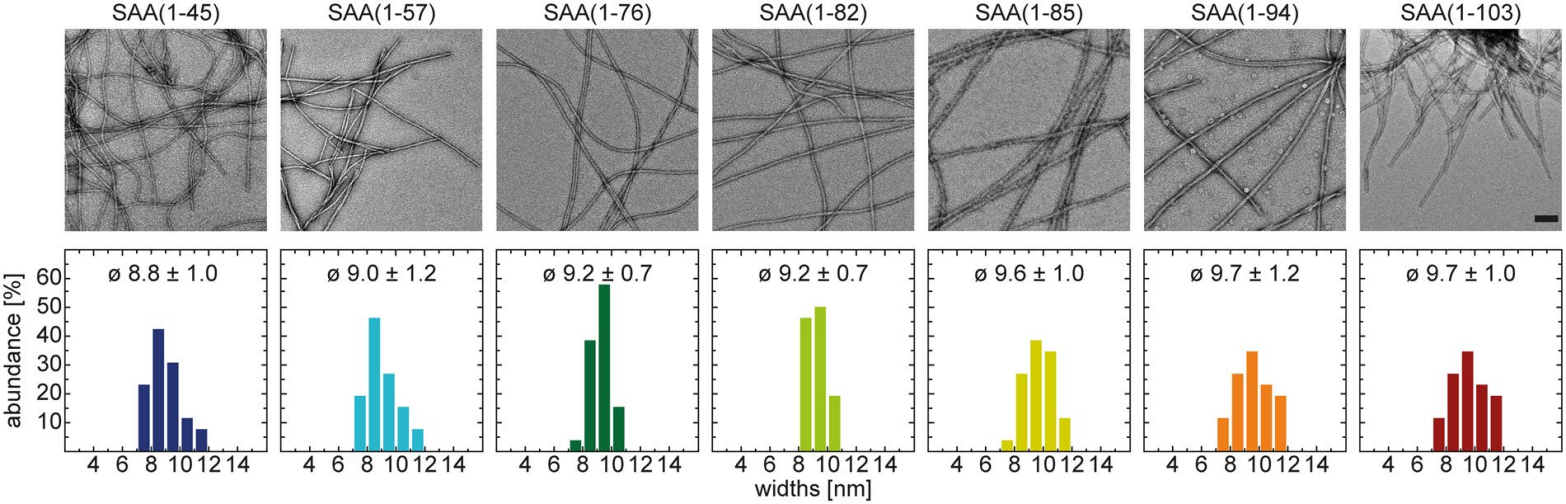
Quantitative analysis of fibrils formed *in vitro* from SAA(1–103) and AA proteins. (**A**) Negative-stain TEM images. Scale bar: 100 nm. (**B**) Distribution, mean value and standard deviation of the widths of respective fibrils (n = 30).

**Figure 3:**
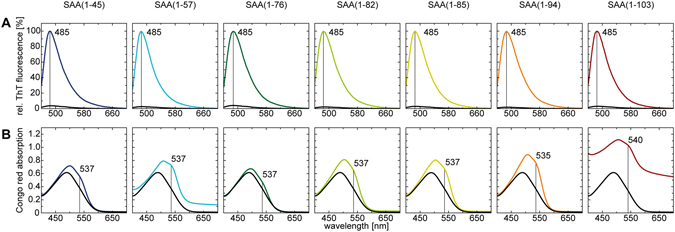
Binding properties of fibrils formed *in vitro* from SAA(1–103) and AA proteins to amyloid specific dyes. (**A**) Normalized ThT fluorescence spectra of ThT without (black) and with fibrils. (**B**) Congo red absorption spectra of pure Congo red solution (black) in comparison to spectra obtained by addition of preformed fibrils.

To examine whether the AA proteins form fibrils *in vitro*, we prepared 80 µM freshly dissolved peptide in Tris buffer (pH 8.0) and incubated the samples for 7 days at 37 °C. After incubation, all solutions gave rise to an increased viscosity, indicating formation of fibril structures. While most samples maintained as transparent solution, SAA(1–103) and SAA(1–57) showed a visibly increased turbidity, caused by macroscopic peptide accumulations in these samples. To confirm and analyze fibril formation we used negative stain transmission electron microscopy (TEM), which revealed in all samples an abundance of long, regular and straight fibril structures (Fig. 2A). While the majority of the fibrils appeared as a flat ribbon without an obvious twist, we also observed a minor population of twisted fibril structures. However, judged from the amount of material visible in TEM fibrils derived from SAA(1–103) and SAA(1–57) occur in locally dense clumps, whereas the other fibril samples seem to be more or less evenly distributed. These differences may derive from the high pIs of SAA(1–103) and SAA(1–57), which notably differ from the other fragments (Table 1), resulting in a lower solubility at near physiological pH. To quantitatively analyze morphological features of the fibrils we measured the distribution of fibril width. The obtained values present a narrow bell-shaped distribution that centers in all cases around 9–10 nm (Fig. 2B). Since all examined fibrils are long and straight, we conclude that fibrils derived from SAA(1–103) and its AA proteins possess a similar morphology without significant differences. In a next step, we examined whether the fibrils exhibit amyloid-like characteristics. First, we tested binding to the amyloid-specific dyes Thioflavin T (ThT) and Congo red (CR) - both typical indicators of amyloid fibrils. The interaction of ThT with fibrils results in an enhanced fluorescence intensity of ThT that can be observed with fluorescence spectroscopy^23^. Binding of CR to amyloid fibrils can be measured by absorption spectroscopy and leads to an increase and a shift to higher wavelength of the absorption maximum of CR^24^. For the measurements we used equimolar amounts of fibrils to ensure quantitative comparability between all samples. We found that SAA(1–103) and all AA fibrils induce a pronounced increase in ThT fluorescence with a maximum at 485 nm (Fig. 3A). We also observed in all samples the typical increase and shift of the absorption maximum of CR to ~540 nm (Fig. 3B). However, due to the turbidity of the fibril solutions of SAA(1–103) and SAA(1–57) a strong baseline shift was observed. In summary, all fibrils showed a clear interaction with both amyloid probes, indicating an amyloid-like structure.

**Figure 4:**
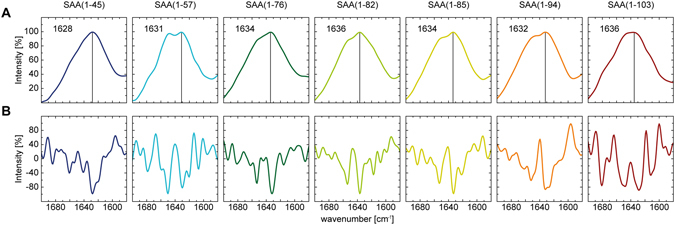
Secondary structure analysis of fibrils formed *in vitro* from SAA(1–103) and AA proteins. (**A**) ATR-FTIR spectra of preformed fibrils with indicated amide I maxima. (**B**) Second derivatives of the ATR-FTIR spectra, which were used to determine the positions of the secondary structural elements for the curve fitting procedure.

Infrared spectroscopy can be used to report the existence of an amyloid-like, aggregated β-sheet conformation^25^. Hence, we examined the fibrils with Fourier transform infrared spectroscopy (FTIR) and found broad bands in the amide I spectral region with maxima centered between 1628–1636 cm^−1^ (Fig. 4A). However, minor differences in the shape of the recorded spectra are evident in the β-sheet region between 1610–1640 cm^−1^ and might indicate differences in the fibril structure. To determine the β-sheet content, we performed a decomposition analysis of the amide I bands. To that end, we applied a curve fit according to the minima of the second derivative (Fig. 4B). We assigned the integrated areas of curves located between 1607 cm^−1^ and 1636 cm^−1^ to β-sheet. Low intensity bands at ~1690 cm^−1^, usually also assigned to β-sheet, were not present (Table 2). We found that all fibrils possess β-sheet contents between 39 und 58%. Strikingly, the highest β-sheet content was detected for fibrils derived from the shortest peptide, while fibrils derived from the longest AA protein exhibit the lowest β-sheet content. Taken together we conclude that SAA(1–103) and all of its AA proteins are amyloidogenic and form amyloid-like fibrils with similar structural properties.

**Table 2:** β-sheet contents of *in vitro* formed fibrils derived from SAA(1–103) and AA proteins, revealed by ATR-FTIR.

Polypeptide	β-sheet content [%]
SAA(1–103)	44
SAA(1–94)	39
SAA(1–85)	52
SAA(1–82)	54
SAA(1–76)	48
SAA(1–57)	45
SAA(1–45)	58

## Discussion

In this study, we have established recombinant expression systems and purification strategies for the production of significant quantities of C-terminal truncated fragments of SAA(1–103) that correspond to naturally occurring AA proteins. Based on that, we have systematically examined the structural features of fibrils derived from these AA proteins.

Previous studies have used fibrils from full-length SAA and certain SAA fragments formed at low pH^18, 26–29^ or physiological pH^20, 21, 30^. Depending on the conditions, these fibrils appear to be twisted or untwisted, curvilinear or straight and show mainly a width distribution of 10–30 nm. However, most of the SAA fragments used in these studies have not been found in diseased tissue. In our study, we showed that all investigated AA proteins form fibrils under nearly physiological pH, leading to mainly non-twisted and straight structures with an average width of 9–10 nm as apparent in TEM (Fig. 2). These values match the average width of amyloid fibrils^1^ and correspond to the numbers reported above. However, amyloid formation depends on the underlying amino acid sequence and environmental conditions such as pH, temperature or salt concentration and morphological heterogeneity of fibrils has been shown for many different proteins between different samples or even within the same sample^31–33^.

The investigated fibrils possess significant β-sheet content as evident from secondary structure analysis with FTIR. We found amide I maxima in the range of 1628–1636 cm^−1^ (Fig. 4). This observation is consistent with previous analysis of fibrils derived from SAA^29^, short N-terminal SAA fragments^28, 29^ or AA amyloidosis cell culture model^26^ reporting high β-sheet content evident from amide I maxima within the range of 1623–1634 cm^−1^. The crystal structures of human SAA1.1 and murine SAA 3 show a high α-helical content without the presence of β-sheet conformation^8, 9^. Furthermore, murine SAA1.1 and SAA1.3 adopt also α-helical structure in solution^10, 20^. In our study none of the fragments investigated show significant α-helical content, demonstrating the importance of the C-terminal tail of murine SAA1.1 for adopting the typical SAA fold^9^ (Fig. 1). Taken together these data indicate that significant conformational changes from α-helical or random coil structure to β-sheet structure take place during fibril formation of SAA or the C-terminal truncated variants.

It has been shown that the N-terminus plays a critical role in amyloid formation of human and murine SAA^17, 18, 20^. All proteins examined in our study comprise an intact N-terminus whereby the smallest fragment tested was SAA(1–45). Together with the fact that all proteins are able to form fibrils with similar morphology (Fig. 2) and amyloid dye binding properties (Fig. 3), it indicates that the fibril core is built up by the N-terminal region of the proteins. However, although the fibrils show comparable amide I maxima in FTIR they vary in other bands of the β-sheet region between 1610 and 1640 cm^−1^ (Fig. 4), indicating differences in the fibril structure. The truncated fragments comprise the same N-terminal amino acid sequence but differ in their C-terminus. This let us suggest that the C-terminus of the truncated fragments might play a modulating role for the fibril structure, which was recently proposed by Jannone *et al*.^34^.

The strong correlation of SAA cleavage sites with clinical variations in tissue distribution of AA amyloidosis in the kidney raises the question whether the C-terminal truncation of the precursor protein contributes to the pathogenesis of the disease. Several studies have proposed specific proteases that degrade SAA, but it is still unknown at which time point the C-terminal truncation takes place^2^. One possible scenario is that proteolytic cleavage of SAA occurs prior to amyloid formation. This is supported by the finding that patients suffering from the “vascular” or “glomerular” form of AA amyloidosis show identical amyloid deposits in other organs, e.g. spleen and kidney^35^. This scenario implies that AA proteins are able to form amyloid fibrils by themselves. They could be generated within the bloodstream or tissue and form fibrils after accumulation in different organs. Another possible scenario is that proteolytic cleavage takes place after amyloid formation^2^. This implies that full-length SAA is able to form fibrils. Furthermore, it implies that AA proteins are either not able to form fibrils by themselves or they are not generated although they possess the intrinsic potential to form fibrils. In our data, we have shown that full-length SAA and all AA proteins are able to form amyloid-like fibrils. This data support the idea of amyloid formation as a pre-fibril-formation event but also shows that truncation of the precursor protein is no prerequisite for amyloid formation and could also occur as post-fibril-formation event.

To elucidate the exact time point of proteolytic cleavage of SAA and its contribution to the pathogenesis of AA amyloidosis further experiments need to be carried out.

## Materials and Methods

### Prediction of amyloid-prone regions

Amyloid-prone regions of murine SAA1.1 were predicted using the programs WALTZ^36^, Foldamyloid^37^, TANGO^38^, AmylPred^39^ and Aggrescan^40^.

### Generation of protein structure homology model

The protein structure homology model was generated using the SWISS-MODEL protein structure homology-modelling server (www.swissmodel.expasy.org). The amino acid sequence of murine SAA1.1 was aligned with the template structure of native human SAA1.1 protein (PDB.4IP9)^9^.

### Recombinant expression and protein purification

Full-length murine SAA1.1 protein was recombinantly expressed with a pMal-c2X vector in *Escherichia coli* RV308 and purified as described previously^26^.Fragments of mSAA have been generated by introduction of stop codons into the mSAA gene at specific positions using QuikChange XL Site-Directed Mutagenesis Kit (Stratagene). Expression of the fragments was performed in *Escherichia coli* Rosetta at 30 °C using ZYP-5052 autoinduction medium. Purification of the fragments has been done in seven steps: (i) amylose resin high flow (New England Biolabs) chromatography, (ii) nickel-Sepharose fast flow (GE Healthcare) chromatography, (iii) fusion protein cleavage by overnight incubation with tobacco etch virus protease at 34 °C, (iv) nickel-Sepharose fast flow chromatography to separate SAA from the fusion protein and maltose-binding protein, (v) Source 15 RPC (GE Healthcare) reversed-phase chromatography, (vi) size exclusion chromatography with a Superdex 75 (GE Healthcare) and (vii) Source 15 RPC (GE Healthcare) reversed-phase chromatography. The purified protein was lyophilized using an alpha 2–4 LDplus freeze dryer (Christ).

### Denaturing protein gel electrophoresis

Samples were prepared by redissolving an aliquot of freeze dried protein in 30 µL water and addition of 10 μL of 4x NuPAGE LDS Sample Buffer (Life Technologies). Samples were heated for 10 min at 95 °C and loaded onto a 4–12% NuPAGE Bis-Tris Gel (17 wells; Life Technologies). The gel was run for 35 min at 180 V in NuPAGE MES-SDS Running Buffer (Life Technologies), stained with a solution containing 30% ethanol absolute, 10% acetic acid and 0.25% Coomassie brilliant blue and destained with a solution containing 30% ethanol absolute and 10% acetic acid.

### Matrix-assisted laser desorption/ionization (MALDI) mass spectrometry (MS)

Spectra were recorded using an Ultraflex-II MALDI TOF/TOF mass spectrometer (Bruker) operated with Flex Control 3.0 software and externally calibrated with a peptide or protein calibration mixture (Bruker). For sample preparation, 1 µL of 2,5-dihydroxybenzoic acid solution (7 mg solved in 100 µL methanol, Bruker) was mixed with 1 µL protein solution. 1 µL of that mixture was deposited onto a stainless steel target.

### Determination of protein concentration

The lyophilized proteins were freshly dissolved in water and the protein concentrations were determined in the stocks by the method of Gill and Hippel. The molar extinction coefficients used were 24750 M^−1^ cm^−1^ for SAA(1–103), 23470 M^−1^ cm^−1^ for SAA(1–94), 20910 M^−1^ cm^−1^ for SAA(1–85), SAA(1–82), SAA(1–76), SAA(1–57) and 15220 M^−1^ cm^−1^ for SAA(1–45)^41^.

### Circular dichroism (CD) measurements

Lyophilised protein was dissolved in water (4 °C or 37 °C) at a concentration of 1 mg/ml. Protein concentration was determined by absorption at 280 nm and diluted to a final concentration of 15 µM peptide in 50 mM sodium phosphate buffer (pH 8, 4 °C or 37 °C). Measurements were done with a J-810 CD spectrometer (Jasco Analytical Instruments) and a 1 mm quartz cuvette (SUPRASIL^®^ 110-QS). CD spectra were recorded using a band width of 1 nm, a scanning speed of 100 nm/min and a temperature of 4 °C or 37 °C. Resulting spectra represent the average of 20 accumulations. The mean residue weight ellipticity *[θ]*_*MRW*_ was calculated using the measured ellipticity *θ*, the mean residue weight *MRW*, cuvette path length *d* and the protein concentration *c* according to formula (1). *MRW* represents the protein molecular weight divided by the number of peptide bonds.

1 $${[\theta ]}_{MRW}=\theta \ast MRW/d\ast c$$

### *In vitro* fibrillation of SAA protein

Proteins were incubated at 80 µM concentration in 50 mM Tris buffer (pH 8.0) in a 1.5 ml reaction tube for 7 days at 37 °C and 300 rpm using a circular shaker (IKA MTS2/4 digital) placed in an incubator (Binder BD53).

### Transmission electron microscopy (TEM)

Specimens were prepared by placing 5 µL of the sample solution onto a formvar and carbon coated 200 mesh copper grid (TED Pella). After incubation for 1 min at room temperature, the excess solution was soaked away and the grid was washed three times with water. Negative staining was performed by washing the grid three times with 2% (w/v) uranyl acetate solution. Grids were examined with a JEM-1400 TEM (JEOL) that was operated at 120 kV. Analysis of fibril morphology has been done with ImageJ software from the electron micrographs.

### Thioflavin T (ThT) fluorescence measurement

Spectra were recorded at room temperature using a LS-55 fluorescence spectrometer (PerkinElmer) and an ultra micro fluorescence cuvette (105.253-QS, Hellma). Samples contained 20 μM ThT, 50 mM Tris buffer (pH 8.0) and 10 μM fibrils and were incubated for 2 min before measurement. Fluorescence emission spectra were recorded from 460 to 700 nm, using an excitation wavelength of 450 nm, excitation and emission slit settings of 7 nm and a scan speed of 100 nm/min.

### Congo red (CR) absorption measurement

Spectra were recorded at room temperature, using a Lambda 35 UV/VIS spectrometer (PerkinElmer). Samples contained 10 µM CR, 50 mM Tris buffer (pH 8.0) and 20 µM fibrils and were incubated for 2 min before measurement. Absorption spectra were recorded from 300 to 700 nm using a slit width of 1 nm and a scan speed of 480 nm/min.

### Attenuated Total Reflectance Fourier Transform Infrared Spectroscopy (ATR-FTIR)

Spectra were recorded at room temperature on a Tensor 27 FTIR spectrometer (Bruker) equipped with a BIO-ATR II cell and a photovoltaic LN-MCT detector cooled with liquid nitrogen. 20–40 µL of the sample (80 µM in 50 mM Tris buffer, pH 8.0) was placed onto the crystal of the BIO-ATR II cell. Spectra represent averages of 64 scans, using an aperture of 4 mm and an instrument resolution of 4 cm^−1^ with four times zero filling. Spectra were baseline corrected by the rubber band method. Positions of the secondary structural elements were chosen according to the second derivative and spectra were fitted with mixed Gaussian-Lorentzian curves by the Levenberg-Marquardt algorithm. Integrated areas of curves located between 1607–1636 cm^−1^ were assigned to β-sheet contents.

### Physico-chemical parameters

Protein Calculator v3.4, the tool developed by Chris Putnam at the Scripps Research Institute (www.scripps.edu/~cdputnam/protcalc) and the Protparam tool at the ExPASy proteomics server of the Swiss Institute of Bioinformatics were used to calculate physico-chemical parameters for SAA(1–103).

### Data Availability

The datasets generated during and/or analysed during the current study are available from the corresponding author on reasonable request.
